# A multilevel Bayesian Markov Chain Monte Carlo Poisson modelling of factors associated with components of antenatal care offered to pregnant women in Nigeria

**DOI:** 10.1186/s12913-023-09710-2

**Published:** 2023-07-05

**Authors:** Omon Stellamaris Fagbamigbe, Olugbenga Sunday Olaseinde, Oluwasomidoyin O. Bello, Vincent Setlhare, Jackline Mosinya Nyaberi, Anthony Ike Wegbom, Ayo Stephen Adebowale, Adeniyi Francis Fagbamigbe

**Affiliations:** 1 Portsmouth Business School, Faculty of Business and Law, University of Portsmouth, Portsmouth, UK; 2 Data insight and analytics, Sopra Steria Limited, Hemel Hempstead, United Kingdom; 3 Department of Sociology, Adekunle Ajasin University, Akungba Akoko, Nigeria; 4 Department of Obstetrics and Gynaecology, College of Medicine, University of Ibadan, Ibadan, Nigeria; 5 Department of Family Medicine and Public Health, Faculty of Medicine, University of Botswana, Gaborone, Botswana; 6 Department of Environmental Health and Disease Control, Jomo Kenyatta University of Agriculture and Technology, Nairobi, Kenya; 7 Department of Public Health Sciences, College of Medical Sciences, Rivers State University, Port Harcourt, Nigeria; 8 Department of Epidemiology and Medical Statistics, College of Medicine, University of Ibadan, Ibadan, Nigeria; 9 Population Health and Research Entity, North-West University, Mafikeng, South Africa; 10 Division of Population and Behavioural Science, School of Medicine, Health Data Science Unit, University of St Andrews, St Andrews, UK; 11 Institute of Applied Health Sciences, School of Medicine, Medical Sciences & Nutrition, University of Aberdeen, Aberdeen, UK; 12 Research Institute for Health & Wellbeing, Coventry University, Coventry, UK

**Keywords:** Antenatal care, Nigeria, WHO guidelines, ANC contacts, Supplements, Quality of care, ANC components

## Abstract

**Background:**

The most recent WHO guideline on antenatal care (ANC) utilization reaffirmed the necessary and compulsory care and services a pregnant woman should receive to maximize the importance and gains of ANC. While most studies focused on the time of initiation and number of ANC contacts, emphasis was rarely placed on the components of ANC offered to women. This study assessed how complete the components of ANC received by pregnant women are as a proxy for the quality of ANC services offered in Nigeria. We also assessed the clustering of the components and state-level differentials and inequalities in the components of ANC received in Nigeria.

**Methods:**

We used nationally representative cross-sectional data from the 2018 Nigeria Demographic Health Survey. We analysed the data of 11,867 women who had at least one ANC contact during the most recent pregnancy within five years preceding the survey. The assessed components were tetanus injection, blood pressure, urine test, blood test, iron supplement, malaria intermittent preventive treatment in pregnancy (IPTp), and told about danger signs. Others are intestinal parasite drugs (IPD)intermittent and HIV/PMTCT counsel. Descriptive statistics, bivariable and multivariable multilevel Bayesian Monte Carlo Poisson models were used.

**Results:**

In all, 94% had blood pressure measured, 91% received tetanus injection, had iron supplement-89%, blood test-87%, urine test-86%, IPTp-24%, danger signs-80%, HIV/PMTC-82% and IPD-22%. The overall prevalence of receiving all 9 components was 5% and highest in Ogun (24%) and lowest in Kebbi state (0.1%). The earlier the initiation of ANC, the higher the number of contacts, and the higher the quality of ANC received. Respondents with higher education have a 4% (adjusted incidence risk ratio (aIRR): 1.04, 95% credible interval (CrI): 1.01–1.09) higher risk of receiving more components of ANC relative to those with no education. The risk of receiving more ANC components was 5% (aIRRR: 1.05, 95% CI: 1.01–1.10) higher among pregnant women aged 40 to 49 years than those aged 15 to 19 years. Women who decide their healthcare utilization alone had a 2% higher risk of getting more components than those whose spouses are the only decision taker of healthcare use. Other significant factors were household wealth status, spouse education, ethnicity, place of ANC, and skill of ANC provider. Pregnant women who had their blood pressure measured were very likely to have blood and urine tests, tetanus injections, iron supplements, and HIV talks.

**Conclusions:**

Only one in every 20 pregnant women received all the 9 ANC components with wide disparities and inequalities across the background characteristics and the States of residence in Nigeria. There is a need to ensure that all pregnant women receive adequate components. Stakeholders should increase supplies, train, and create awareness among ANC providers and pregnant women in particular.

**Supplementary Information:**

The online version contains supplementary material available at 10.1186/s12913-023-09710-2.

## Introduction

Although improvements have been recorded in maternal and child health globally, most developing countries in sub-Saharan Africa (SSA) have continued to lag in the pace of this improvement [1]. Most maternal and child health indicators put the Low and Middle-Income Countries (LMIC) including Nigeria at the lower end of the scale [1–3]. The Maternal and Child Health (MCH) situation in the SSA remained worrisome when compared to the advanced countries and some other developing countries. A childbearing woman in developed countries has only 1 in 5,400 chance of dying from pregnancy and childbirth-related complications compared with only 1 in 22 chances in SSA [1].

To facilitate the attainment of Sustainable Development Goals (SDGs) on improved health and wellbeing [4], in its Strategy for Health 2016 to 2030, the UNICEF envisioned “a world where no child dies from a preventable cause and all children reach their full potential in health and wellbeing” [5]. Efforts focused on achieving this vision birthed the goal to “end preventable maternal, newborn and child deaths” [5]. This underscores the need to ensure guaranteed maternal and child health without inequality at any level. Antenatal care (ANC) is recognised worldwide as the main approach to ensure safe maternal and infant health outcomes. Compliance with ANC protocol is important in the management of pregnancy conditions and is essential for safe delivery [6–9]. The standards in the existing ANC protocol were reviewed in 2016 to reinvigorate the ANC program to guarantee the realization of the relevant goal of the SDGs by 2030 [9]. The reviewed WHO guideline on ANC utilization established three main pillars that can help maximize the gains and importance of ANC utilization. They are (i) initiation of first ANC contact in the first trimester of gestation (ii) having a minimum of 8 ANC contacts and (iii) receiving all components of ANC during the contacts with ANC providers. Therefore, it is not just sufficient to initiate ANC early and have at least 8 ANC contacts, a pregnant woman must receive all the components of ANC services. WHO therefore prescribed the components of ANC to achieve effective results all over the world [9]. Thus, among other reviewed standards, several services were packaged to make up for the minimum quality of ANC available to a pregnant woman. Emphasis was laid on the compulsory administration of specific components of ANC [9]. WHO identified and grouped the components to ensure standard quality of ANC into three (i) history taking, assessment, physical examination and laboratory tests, (ii) health promotion and education on nutrition, delivery, danger signs, and child health and (iii) care provisions such as tetanus toxoid immunization, and others [9–12]. The specifics of these components vary slightly across countries.

Although evidence shows that ANC coverage has increased in Nigeria [13–16], improvement in coverage alone cannot guarantee safe maternal and infant health outcomes [17, 18]. A Nigerian study showed evidence of an unacceptable quality of ANC with only about 1 in 8 pregnant women who had ANC contacts being provided with the minimum quality standard [17]. The study further emphasized that the poor quality of ANC among childbearing women might have contributed to the non-realisation of the defunct Millennium Development Goals (MDGs) on child and maternal health in Nigeria.

Early initiation of ANC, a sufficient number of ANC contacts, attention by skilled health workers, living in urban areas, better education, short distance to health facilities, media exposure, and household wealth quintile have been associated with the quality of ANC received by pregnant women in Nigeria [2, 17, 19–21]. Older pregnant women, higher parity, higher level of education, and higher household wealth status were identified as factors associated with receiving a good quality of ANC by a similar study in Nepal [20]. Also, a Kenyan study pointed out that the level of women’s literacy, employment status, and receiving all ANC services from a health facility had a relationship with having a good quality of ANC by pregnant women [22, 23]. Studies have identified that the commonest components of ANC services received by pregnant women were blood pressure measurement, iron supplement, and urine test [17, 22]; while education on PMTCT was low in Nigeria as only 2 in 5 tested for HIV during ANC visit [17] and negligible proportion had an ultrasound performed.

While most studies focused on the time of initiation of ANC contacts and the minimum number of ANC contacts made, such emphasis was rarely placed on the components of ANC offered to women during the ANC contacts. Whereas, up-to-date information on the components of ANC services could provide a good understanding of the level of compliance with the recommended components and standards of ANC in Nigeria and could provide relevant evidence-based information necessary to inform policy on the implementation of the WHO ANC guidelines. This study is also a response to the United Nations' call for periodical monitoring and evaluation of how close countries of the world are to attaining the specific targets of the Sustainable Development Goals (SDG) [4]. The call also encouraged sub-country analysis. Our study shows how close Nigeria is to SDG-3 target 3.1: “By 2030, end preventable deaths of newborns and children under 5 years of age, with all countries aiming to reduce neonatal mortality to at least as low as 12 per 1,000 live births and under-5 mortality to at least as low as 25 per 1,000 live births” [4]. The services received during ANC contacts are of paramount importance to the attainment of SDG-3. Therefore, this study assessed the number of components of ANC received by pregnant women in Nigeria and its associated factors. We also assessed state-level differentials and inequalities in the quality of ANC care across the states in Nigeria and examined the clustering of the ANC components (or its lack) to inform policy intervention and research translation. The study provided recommendations on the pathways for the improvement of maternal and child health outcomes in Nigeria.

### Conceptual framework

Having ANC components could be influenced by different factors. These range from the personal characteristics of the individuals and the particular household they come from to the peculiarity of their communities and states of residence. Put together, women and their household compositional factors as well as the contextual factors based on community and state of residence and the overall health system could influence the components of ANC offered and received. Of importance are the community factors (such as poverty, illiteracy, rural residence, and poor media access and societal factors such as religion and ethnicity as they could affect the uptake of components. Based on existing literature [17, 19, 22–25], the important individual-level factors are maternal age, education, employment status, access to media, household wealth tertiles, women's autonomy, birth interval, birth order, children ever born, current marital status, place of residence, religion, and ethnicity, family mobility, household headship, health insurance coverage. The community-level factors include community poverty, unemployment, illiteracy, and media access rates as well as state-level rural population percentage.

### Methodologies

#### Study setting and data source

**Fig. 1:**
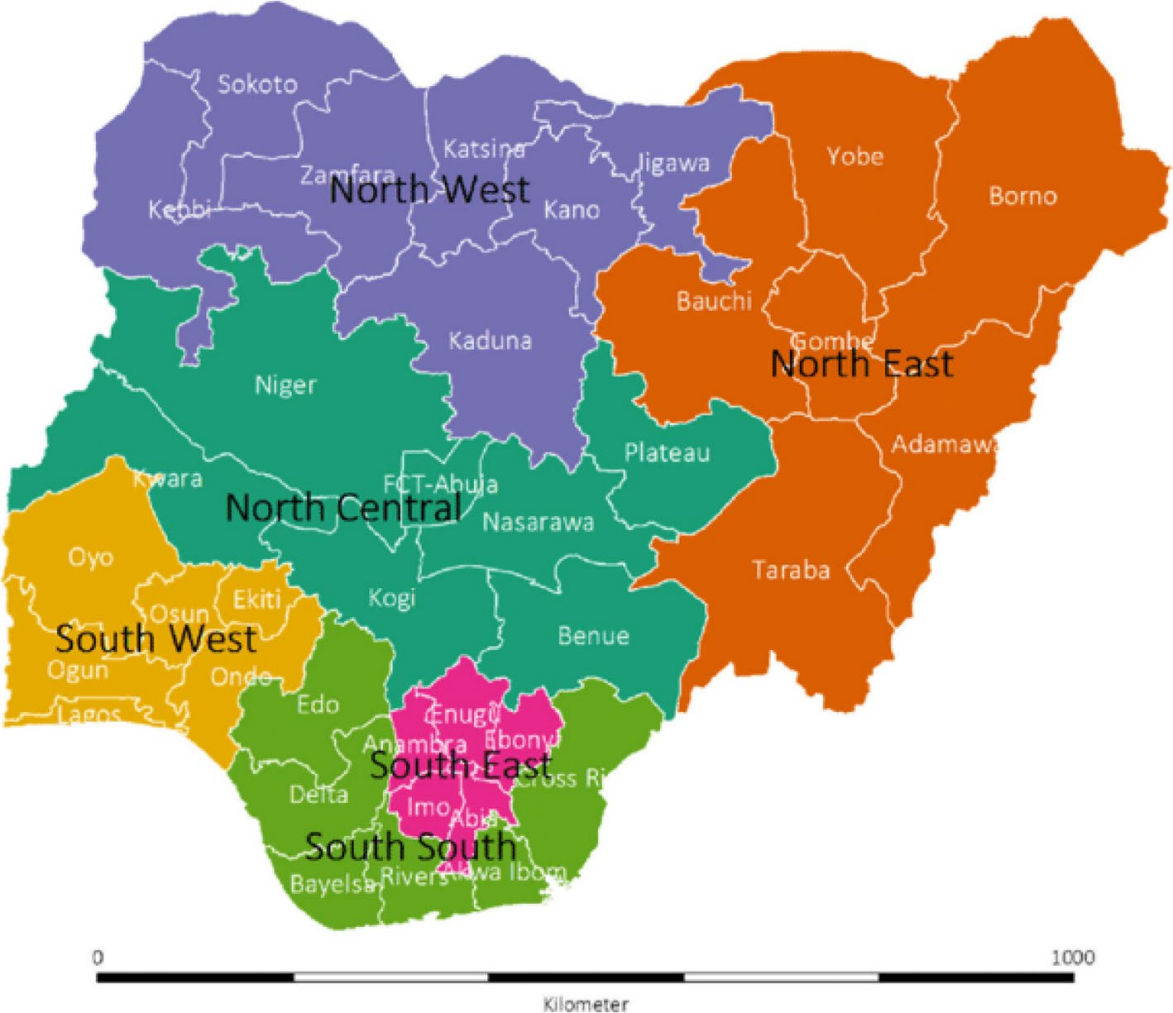
Map of Nigeria showing the 36 states and the federal capital territory (FCT), by the geopolitical regions/zones

The study was retrospective and cross-sectional in design. Data were extracted from the nationally representative 2018 Nigeria Demographic Health Survey (NDHS) conducted by ICF Macro Calverton, Maryland, USA in conjunction with the Nigeria National Population Commission (NPC), Nigeria [15]. Administratively, Nigeria is divided into 36 states and the Federal Capital Territory (FCT). The states are further grouped into 6 regions as shown in Fig. 1. Although the regions have no administrative functions, people within each region are deemed to have similar characteristics, culture, ethnicity, vegetation etc. Each state is subdivided into local government areas (LGAs). Using the most recent 2006 Population Census, each LGA was subdivided into convenient areas called census enumeration areas (EAs).

### Sampling techniques

A two-stage probability sampling was adopted to select the respondents who were women of childbearing age (15–49). The EAs were the primary sampling unit (PSU). In each state, depending on the population, about 38 EAs were selected with probability proportional to EA size making a total of 1,400 EAs in the first stage. Then a full list of households in all selected EAs in the first stage was drawn to serve as the sampling frame for household selection in the second stage. In the second stage, 30 households were selected in every EAs using equal probability systematic sampling. All eligible women of reproductive age (15–49 years) in each selected household were interviewed. Due to the non-proportional allocation of sample sizes at the different states, LGAs and EAs, as well as the possible differences in response rates, sampling weights were applied in all the descriptive analyses. A total of 41,821 women aged 15–49 years were interviewed [15].

### The data

All respondents (women aged 15–49 years) were asked if they had any pregnancy or birth within 5 years preceding the survey (2013 to 2018). Those who had at least one birth were thereafter asked questions relating to conception, and pregnancy-related care including ANC visits made, time ANC began, ANC care provider, etc. of each birth delivery starting from the most recent. Our analysis is based on the information provided on the most recent deliveries and the associated pregnancy information. We assessed all pregnancy history between 2013 and 2018. Among the 21,785 women who provided information about the ANC visits during their most recent pregnancy within the five years that preceded the survey, 16,448 (75.5%) made at least one ANC visit. Furthermore, of those that had at least one ANC contact, only 11,867 (72.1%) provided the ANC components received during all ANC contacts for the most recent pregnancy. Our analysis was based on the components of ANC received by the 11,867 women.

### Multilevel structure of the data

We identified three distinct levels: individual, community and state levels. The likelihood of a woman receiving a certain number of ANC components may be influenced by her background characteristics and her household characteristics (Level 1). The community (Level 2) and the state (Level 3) where a woman resides in Nigeria may also influence receiving ANC components. The “communities” are synonymous with “EAs”, “neighbourhoods” and “clusters”. The community behaviour could be informed by literacy level as well as other sociocultural factors. The states in Nigeria varied extensively in terms of the availability of resources, and rural–urban population. In all, there were 11,867 individuals at level 1, 418 communities at level 2 and 37 states at level 3.

### Variables

The outcome variable in this study is the quality of ANC services using the number of ANC components received as a proxy. The identified components were receiving tetanus injection, intestinal parasite drugs (IPD), blood pressure, urine test, blood test, iron supplement, three or more doses of malaria intermittent preventive treatment in pregnancy (IPTp), being told about danger signs, and counselled on HIV/PMTCT (either talked about HIV transmitted mother to child, things to do to prevent getting HIV, getting tested for HIV). These components have been recommended and recognized in the literature [9, 10, 17, 20, 22, 26].

The independent variables include Level 1: maternal age (15–19,20–24, 25–29, 30–39, 40–49 years), education (no education, primary, secondary and higher), spouse education (no education, primary, secondary and higher), employment status (currently employed or not), spouse employment status (currently employed or not), access to media (at least one of radio, television, or newspaper), household wealth tertiles (lowest, middle, and highest), women's autonomy using “who decides respondents health care” (respondent alone, respondent/spouse, and spouse alone) as a proxy. Others are birth interval (firstborn, < 36 months, and >  = 36 months), birth order (1, 2, 3, 4 and 5 +), children ever born (none, 1–2, 3–4, 4 +), current marital status (currently married or living together, divorced/separated/widowed, never married), religion (Islam, Christian, others), and ethnicity (Hausa/Fulani, Igbo, Yoruba, and others). Family mobility (had stayed less than five years at residence or not), wanted child when became pregnant (then, later or not more), household headship (male or female), health insurance coverage (yes or no), place of ANC (health institution or non-health institution such as missions, homes and traditional attendants) and ANC caregiver (skilled or unskilled). Level 2: the place of residence (rural/urban), the poverty rate (high or low), unemployment rate (high or low), illiteracy rate (high or low), and media access rate (high or low). We computed the community socioeconomic (SES) disadvantage composite score using principal component analysis of the proportion of respondents within each community with rural dwellers, no media access, illiterates, poor, and unemployed. The SES was categorized into three tertiles: lowest, middle, and highest. Level 3: proportion of the rural population in the states of residence. It was categorised as low rural proportion (0% to 33.3%); an average rural proportion (33.4% to 66.7%) and a high rural proportion (66.8% to 100%).

### Statistical methods

Data were analysed using descriptive statistics, bivariable, and multivariable logistic regression using STATA version 16 (Stata Corp, Texas, USA). We invoked the “SVY” command in STATA to adjust for the study design and the sampling weights. Frequency tables showing percentages were used to describe the distribution of study respondents’ characteristics and the distribution of outcome variables by the respondents’ characteristics (Table 1).

**Table 1:** Respondents’ characteristics and having all ANC components during the most recent pregnancy

**Characteristics**	**n (%)**	**Received All 9**	
		**%**	*p-value
**Age of mothers(years)**			**0.004**
15 – 19	705(6.0)	2.0	
20 – 24	2569(21.7)	4.1	
25 – 29	3314(27.9)	5.3	
30 – 39	4420(37.2)	6.0	
40 – 49	856(7.2)	5.4	
**Highest educational level**			**0.000**
No education	4057(34.2)	1.6	
Primary	1902(16.0)	5.2	
Secondary	4587(38.7)	6.9	
Higher	1320(11.1)	9.6	
**Spouse’s highest education**			**0.000**
No education	2828(25.3)	1.1	
Primary	1631(14.6)	5.0	
Secondary	4494(40.2)	6.4	
Higher	2209(19.7)	7.3	
**Who decides respondent’s healthcare**			**0.000**
Respondent alone	1176(10.5)	6.6	
Respondent and Spouse	3937(35.1)	7.0	
Spouse alone	6093(54.4)	3.4	
**Media exposure**			**0.000**
Unexposed to media	3519(29.7)	1.9	
Exposed to media	8348(70.3)	6.5	
**Ethnicity**			**0.000**
Hausa/Fulani	4668(39.3)	1.6	
Yoruba	1680(14.2)	12.9	
Igbo	2050(17.3)	8.5	
Others	3468(29.2)	4.1	
**Religion**			**0.000**
Islam	6750(56.9)	3.4	
Other Christians	5064(42.6)	7.6	
Others	53(0.5)	0.0	
**Marital Status**			0.385
Never married	267(2.3)	10.4	
Living with a spouse	11,280(95.1)	5.0	
Widowed/Divorced/Separated	320(2.7)	6.4	
**Household Wealth status**			**0.000**
Lowest	2834(23.9)	1.7	
Middle	3925(33.1)	4.0	
Richest	5107(43.0)	9.0	
**Children ever-born**			**0.001**
1 or 2 births	4260(38.9)	6.0	
3 or 4 births	3424(28.9)	6.2	
More than 4 births	3822(32.2)	3.2	
**Birth order**			**0.000**
First	2315(19.5)	6.6	
Second	2305(19.4)	5.4	
Third	1903(16.0)	6.4	
Fourth	1521(12.8)	5.9	
Fifth or higher	3822(32.2)	3.2	
**Birth interval**			**0.001**
First birth	2315(19.5)	6.6	
< 36 months	5520(46.6)	4.1	
> = 36 months	4013(33.9)	5.8	
**Current employment status**			**0.000**
Employed	8346(70.3)	6.1	
Unemployed	3521(29.7)	2.9	
**Spouse current employment statement**			**0.000**
Employed	8846(74.5)	5.9	
Unemployed	3021(25.5)	2.8	
**Sex of the Household Head**			0.110
Male	10,716(90.3)	5.0	
Female	1151(9.7)	6.3	
**Wanted Last child**			0.738
Then	10,338(87.1)	5.1	
Later	1177(9.9)	5.4	
Never	352(3.0)	4.1	
**Family Mobility**			**0.019**
5 + yr	9561(80.6)	4.9	
Less stable 0-4 yr	2305(19.4)	6.3	
**Have health Insurance**			0.223
No	11,563(97.4)	5.1	
Yes	304(2.6)	7.0	
**Place of antenatal care**			**0.026**
Non-health facility	439(3.7)	1.9	
Health facility	11,428(96.3)	5.3	
**Provider of ANC assistance**			**0.000**
Unskilled provider	1724(14.5)	1.5	
Skilled provider	10,143(85.5)	5.7	
**No of ANC Visits**			**0.000**
1–3	2859(24.4)	2.0	
4–7	5960(51.0)	4.8	
8 +	2872(24.6)	7.6	
**First ANC Visit**			**0.000**
Trimester 1	2805(23.7)	8.2	
Trimester 2	7432(62.7)	4.6	
Trimester 3	1611(13.6)	2.4	
**Region**			**0.000**
North central	1420(12.0)	4.5	
North East	2143(18.1)	3.1	
North West	3770(31.8)	1.5	
South East	1478(12.5)	8.3	
South South	1112(9.4)	5.8	
South West	1945(16.4)	12.2	
**Residence**			**0.000**
Urban	5458(46.0)	6.6	
Rural	6409(54.0)	3.9	
**Community Poverty Rate**			**0.001**
High	4863(41.0)	3.7	
Low	7004(59.0)	5.8	
**Community Illiteracy**			**0.000**
High	4967(41.9)	3.3	
Low	6900(58.1)	5.9	
**Community Unemployment**			**0.007**
High	5226(44.0)	4.1	
Low	6641(56.0)	5.8	
**Community Media barrier**			0.167
High	5817(49.0)	4.6	
Low	6049(51.0)	5.5	
**Community SES Disadvantage**			**0.000**
Lowest	4807(40.5)	9.7	
Middle	4296(36.2)	4.0	
Highest	2764(23.3)	1.3	
**Total**	11,867	5.1	

^*^Significance at χ^2^ 0.05

We fitted a multilevel Bayesian Markov Chain Monte Carlo (MCMC) Poisson-based Generalized Linear Model (GLMs) to the data, with women nested within EAs and the EAs nested within the states. The models have mixed outcomes consisting of fixed and random parts as shown in Eq. (1).

1 $$log\left(\gamma_{ijk}\right)=\underbrace{\beta_0+\sum\nolimits^t_{p=1}\beta_pX_{pijk}}_{Fixed}+\underbrace{U_{0jk}+V_{0k}}_{Random}$$

The “risk” that pregnant woman $i$ of community $j$ from state $k$ will receive ANC component is denoted by ${\gamma }_{ijk}$, ${\mathrm{U}}_{ojk}$ is the random effect of mothers community $j$ in state $k$ and ${\mathrm{V}}_{ok}$ is the random effect of state $k$, ${e}_{ijk}$ is the noise such that ${e}_{ijk}\sim N\left(0,{\sigma }_{e}^{2}\right)$, ${U}_{ojk}\sim N\left(0,{\sigma }_{U}^{2}\right)$ and ${V}_{ok}\sim N\left(0,{\sigma }_{V}^{2}\right)$ in a model with $t$ covariates.

We reported the measure of the rates of receiving the components as incidence rate ratios (IRRs) with their 95% credible intervals (CrI). Measures of variations were explored using the intraclass correlation (ICC) and median incidence rate ratios (MIRR) [27, 28]. The ICCs, an equivalent of the variance partition coefficient (VPC), is the percentage of the total variance in the risk of a pregnant woman obtaining the components that are related to the community and state where they live (i.e. a measure of clustering of the “risk” receiving ANC components in the same community and state). We also estimated the proportion of total variance which are accounted for at the community $\left[{\sigma }_{U}^{2}/\left({\sigma }_{U}^{2}+{\sigma }_{V}^{2}+{\sigma }_{e}^{2}\right)\right]$ and the state $\left[{\sigma }_{V}^{2}/\left({\sigma }_{U}^{2}+{\sigma }_{V}^{2}+{\sigma }_{e}^{2}\right)\right]$ levels. The MIRR is the estimate of the probability that a pregnant woman will receive additional components attributable to the community and state context. The Bayesian MCMC Multilevel Poisson model was implemented using MLwin v3.03 and implemented in Stata V16 with the following parameters: Burnin = 5000; Chain = 50,000, Thinning = 50.

At the bivariable level, we identified all independent variables that were significant at *p* < 0.20 and used them as candidate variables in the multivariable model. We then estimated the adjusted odds ratios of characteristics associated with the components of ANC services. The “collin” command in Stata was used to identify collinear variables and the associated Variance Inflation Factor (VIF). Based on the VIF, the less important pair of collinear variables was dropped from the multiple regression. In the Bayesian MCMC Multilevel Poisson model, we stated the respective levels: levels 1, 2 and 3. We started with a null model (no independent variable), then individual-level alone, then community-level alone, then state-level alone and finally the model. These models were compared using Bayesian Information Criteria (BIC). These were summarised in the supplementary Table A. We used the principal component (PCA) and factor analysis procedure to explore clustering among the components. The PCA method maintains all theoretically relevant variables and avoids the negative influence of high inter-correlation among the variables [29, 30]. The “cluster single-linkage” and “loadingplot” commands in Stata were used to analyse the data and visualize the clustering respectively.

### Ethics approval

This study was based on the analysis of existing survey data. The Institutional Review Board (IRB) of Inner-City Fund International (ICF) Macro at Fairfax, Virginia in the USA reviewed and approved the protocol for the Demographic and Health Surveys Project Phase III (Number FWA000008450). The 2010–2018 DHS’s were categorized under that approval. The IRB of ICF Macro complied with the United States Department of Health and Human Services requirements for the “Protection of Human Subjects” (45 CFR 46). The specific ethical guidelines for the conduct of the study by the IRB on benevolence, non-maleficence and confidentially were followed strictly. Written informed consent was obtained from every study participant before participation and all information was collected without identifiers and kept confidential. We were granted full access to use the data by ICF with authorisation letter 144,644 but we are not allowed to share the data. All intending users are required to request from the original data owners at http://dhsprogram.com.

## Results

**Fig. 2:**
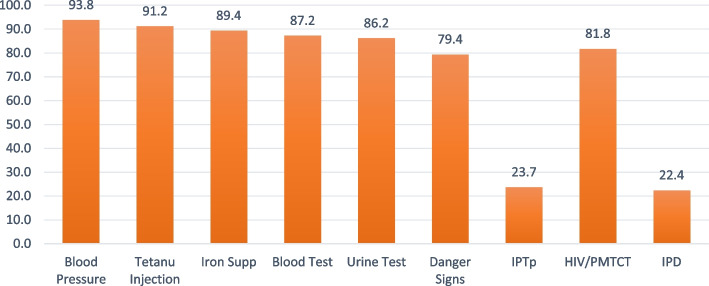
Distribution of ANC components received during the most recent pregnancy in Nigeria

The data was collected from a cross-section of women selected using two-stage probability sampling across the 37 states in Nigeria. In all, 11,867 women provided information about the ANC components received during ANC visits for their most recent pregnancy during the 5 years preceding the survey. According to the owners of the data, in the households interviewed, 42,121 women aged 15–49 years were identified for individual interviews; interviews were completed with 41,821 women, yielding a response rate of 99.3% with 99.2% in urban areas and 99.4% in the rural areas [15]. However, we do not have any information on the population composition of pregnant women in Nigeria. The availability of this would have enabled the assessment of the level of representativeness of the respondents. During the last pregnancy, 94% had their blood pressure measured, nine of every ten women (91%) received a tetanus injection, 89% were given an iron supplement, 87% had a blood test, 86% had a urine test, 82% received counselling on HIV/PMTC, 80% were told about danger signs while only 24% got IPTp and only 22% received IPD (Fig. 2).

**Fig. 3:**
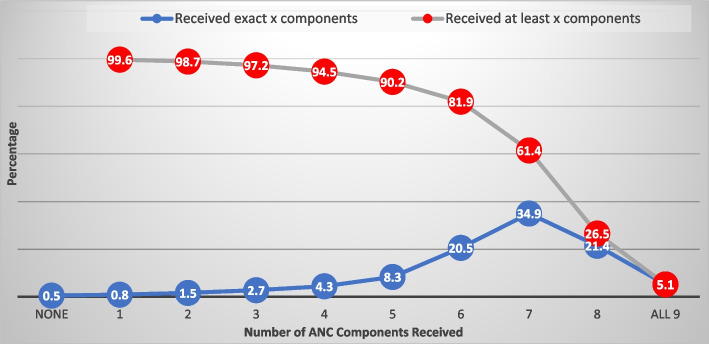
Distribution of ANC components received during pregnancy

Figure 3 shows that only 5% of the respondents received all 9 components (Fig. 3). The commonest (35%) number of components received was 7. About 61% and 90% received at least 7 and at least 5 components respectively.

Table 1 shows the distribution of the respondents by their background characteristics and the prevalence of receiving all the 9 studied components. The prevalence of having the 9 components was highest (6%) among women aged 30 to 39 years compared with 2% among those aged 15 to 19 years. Nearly a quarter (10%) of the pregnant women with higher education who had at least one ANC contact received all the 9 ANC components while less than 2% of those with no education did. The proportion of all the 9 ANC components was lowest among women whose spouse alone decide health care access (3%), no media exposure (2%), Hausa/Fulani (2%), from households in the lowest wealth tertile (2%), North East (3%) North West (2%) and rural areas (4%) as shown in Table 1. All the explanatory variables considered were significantly (*p* < 0.001) associated with receiving all the ANC components received except whether the pregnancy was wanted or not and the type of ANC facility attended. Also, all characteristics in Table 1 were associated with having all 9 components, except marital status, sex of household head, whether the pregnancy was wanted or not, health insurance, and community media access. Having all the components was significantly higher among women from communities with low rates of illiteracy, poverty, and community socioeconomic disadvantage.

**Fig. 4:**
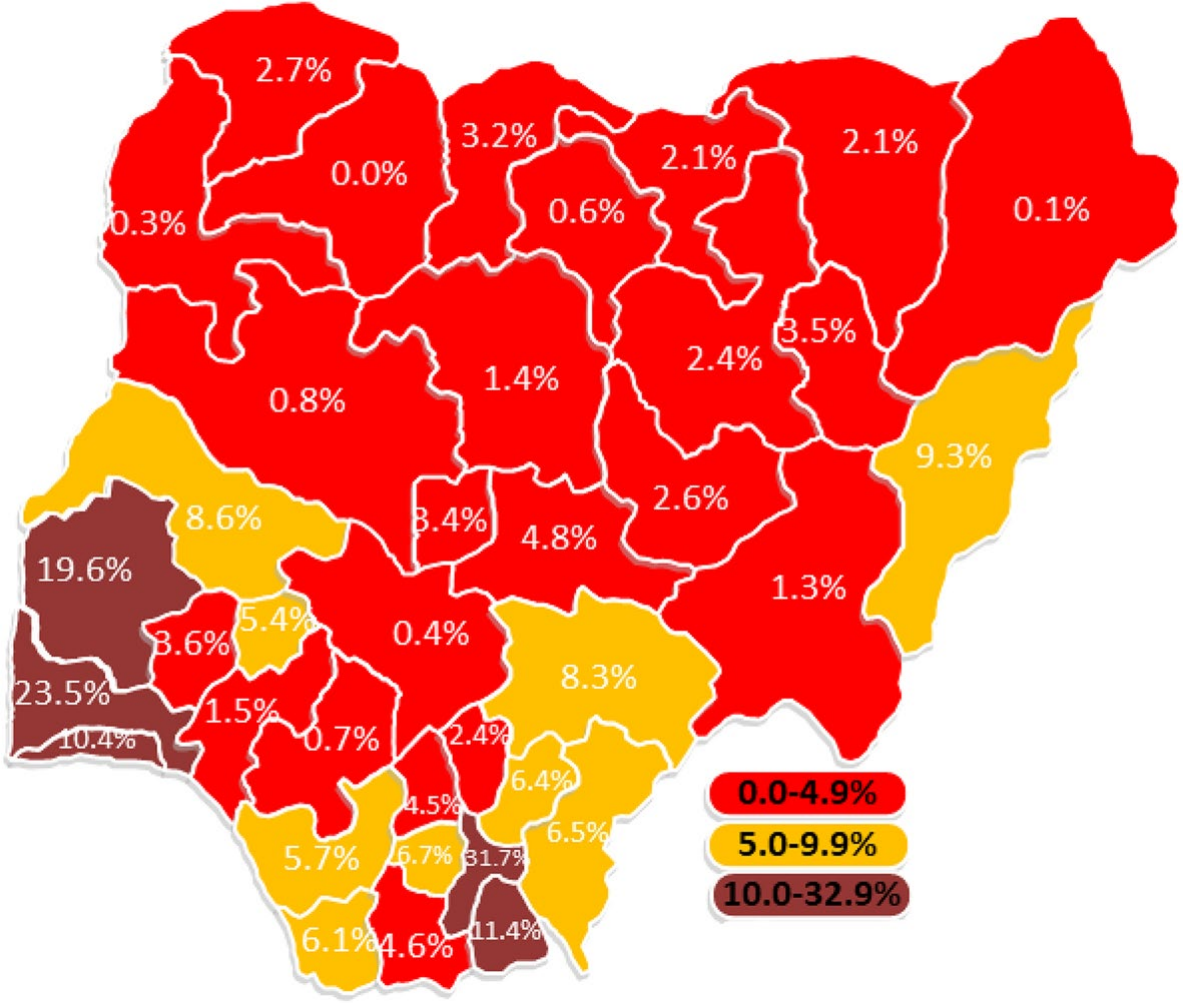
Distribution of the percentages of pregnant women offered all the 9 components of ANC during the most recent pregnancy by States in Nigeria

Figure 4 shows the map of Nigeria with the geographical distribution of the percentage of ANC attendees that received all nine components across the states. The highest prevalence of receiving all the 9 studied components was highest in Abia (32%), none (0%) in Zamfara, Borno (0.1%) and Kebbi (0.3%). Benue (8.3%), Kwara (8.6%) and Adamawa (9.3%) are the only states in the North with at least 5% women receiving all 9 components. The only five states with 10% or higher were from the southern part of Nigeria. The distribution of having all ANC components received during the most recent pregnancy by States and regions in Nigeria is shown in Supplementary Table A.

### Relationship between the number of ANC contacts, the timing of first ANC contact, and receiving all 9 ANC components received in Nigeria

**Fig. 5:**
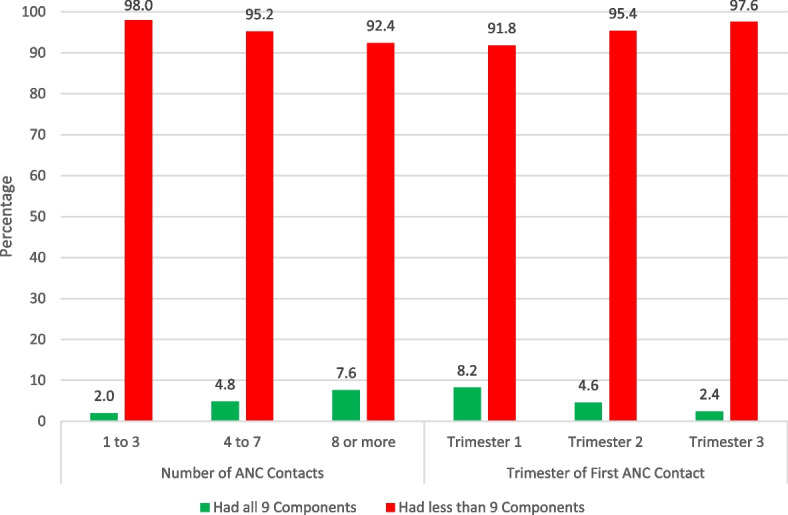
Relationship between the number of ANC visits, the timing of the first ANC visit, and receiving the 9 ANC components during the most recent pregnancy in Nigeria

The number of ANC contacts made during the last pregnancy and the timing of the first ANC contact were both significantly associated with the adequacy of ANC components received (Table 1 and Fig. 5). Figure 5 shows that a linear association exists between the number of ANC contacts made, the timing of the first ANC contacts, and the adequacy of ANC care received. Pregnant women who had first ANC contact during the first trimester had a higher (8%) prevalence of having all 9 components than those who started in the third trimester (2%). Similarly, pregnant women who had 8 or more ANC contact had a higher (8%) prevalence of having all 9 components than those who had less than 4 contacts (2%).

### Factors associated with the number of ANC components received

**Fig. 6:**
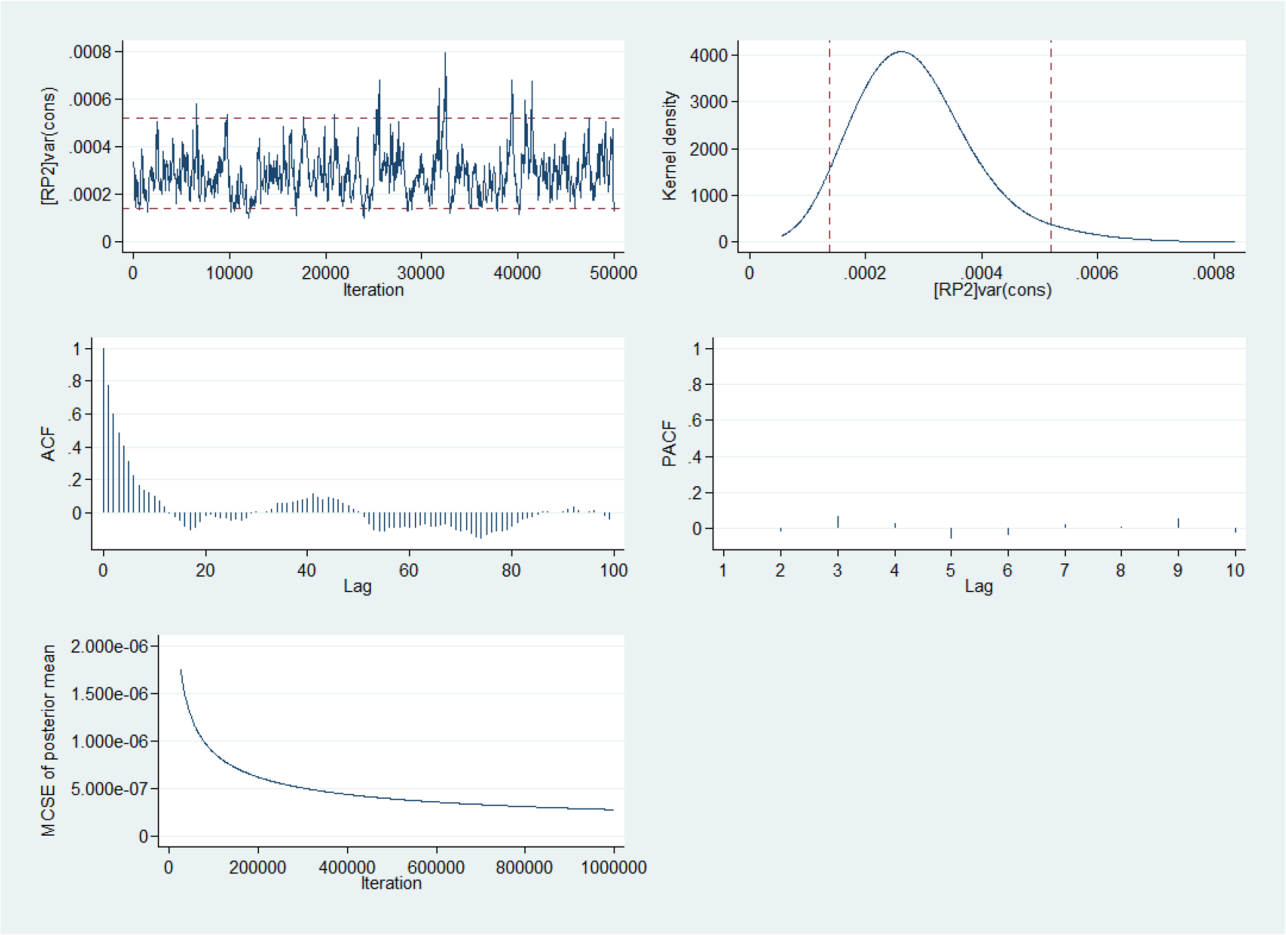
Five-way MCMC graphical diagnostics of the model at the community level

**Fig. 7:**
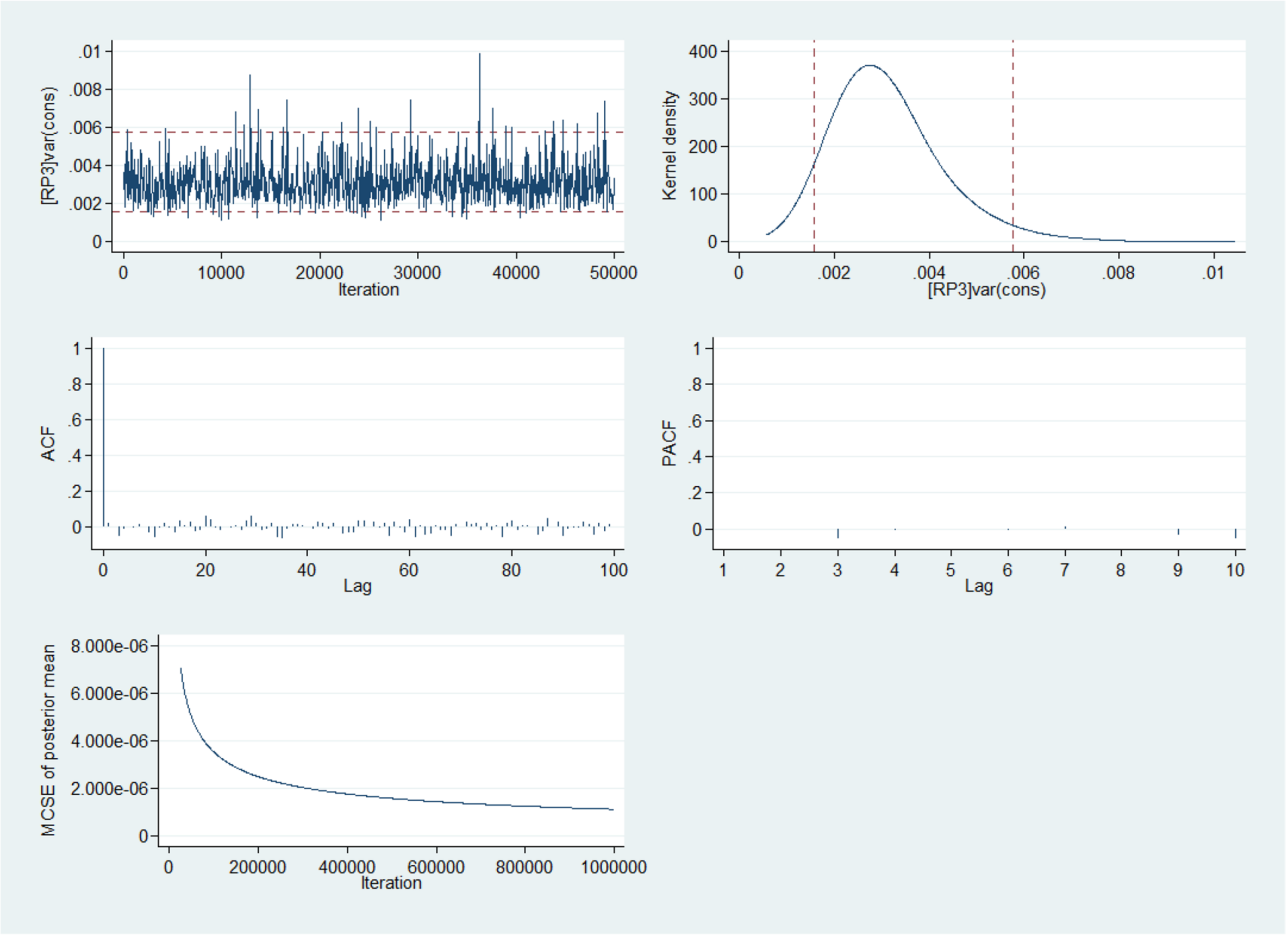
Five-way MCMC graphical diagnostics of the model at the state level

Table 2 shows the crude and adjusted risk of receiving additional components of ANC among the respondents from the multilevel Bayesian MCMC Poisson model. The Bayesian information criteria for model 5 for the lowest among all the five models considered. The five-way MCMC graphical diagnostics of model 5 at the community and state levels are presented in Fig. 6 and Fig. 7 respectively. The Figures show the convergence of the adjusted model presented in Table 2. The Figures gave credence that the adopted model converged and fit for predicting the number of ANC quality received by the women. Respondents with higher education have a 4% (adjusted incidence risk ratio (aIRR): 1.04, 95% confidence interval (CI): 1.01–1.09) higher risk of receiving more components of ANC relative to those with no education (Table 2). The risk of receiving more ANC components was 5% (aIRR: 1.05, 95% CI: 1.01–1.10) higher among pregnant women aged 40 to 49 years than those aged 15 to 19 years. Women who decide their healthcare utilization alone had a 2% higher risk of getting more components than those whose spouses are the only decision taker of healthcare use. Pregnant women from in South East were 6% more likely to receive a higher number of components of ANC relative to those in the North West. Also, respondents from the households in the richest wealth quartile were 3% more likely to receive a higher number of components of ANC relative to those from households in the low wealth category. The risk of having a higher number of components was 4% higher in communities with the lowest SES disadvantaged than those with the highest disadvantage. Other significant variables with having a higher number of components of ANC are spouse education, ethnicity, religion, respondent’s current employment status, family mobility, having health insurance, place of ANC, the skill of ANC provider, and the proportion of the rural population in the states of residence.

**Table 2:** Crude and Adjusted correlates of the number of ANC components received during the most recent pregnancy (NDHS 2018)

**Characteristics**	IRR(95% CI)	significance	aIRR(95% CI)	significance
**Fixed Effects**				
**Age of mothers**				
15 – 19	Reference			
20 – 24	1.05(1.02–1.09)	**0.003**	1.03(0.98–1.07)	0.096
25 – 29	1.09(1.05–1.13)	**0.000**	1.04(0.99–1.09)	**0.045**
30 – 39	1.09(1.06–1.13)	**0.000**	1.04(1.01–1.09)	**0.038**
40 – 49	1.08(1.03–1.12)	**0.001**	1.05(1.01–1.10)	0.045
**Highest educational level**				
No education	Reference			
Primary	1.06(1.04–1.09)	**0.000**	1.01(0.98–1.04)	0.300
Secondary	1.14(1.12–1.16)	**0.000**	1.04(1.01–1.07)	**0.010**
Higher	1.20(1.17–1.23)	**0.000**	1.04(1.01–1.09)	**0.024**
**Spouse’s highest education**				
No education	Reference			
Primary	1.08(1.05–1.11)	**0.000**	1.03(1.01–1.05)	**0.049**
Secondary	1.15(1.12–1.17)	**0.000**	1.05(1.02–1.08)	**0.000**
Higher	1.18(1.15–1.21)	**0.000**	1.05(1.02–1.08)	**0.000**
**Who decides respondent’s healthcare**				
Respondent alone	1.05(1.02–1.07)	**0.001**	1.02(1.01–1.03)	**0.048**
Respondent and Spouse	1.06(1.04–1.08)	**0.000**	1.00(0.98–1.02)	0.454
Spouse alone	Reference			
**Media exposure**				
Exposed to media	1.13(1.11–1.15)	**0.000**	1.03(1.01–1.05)	**0.002**
**Ethnicity**				
Hausa/Fulani	Reference			
Yoruba	1.13(1.10–1.16)	**0.000**	1.03(1.01–1.07)	0.315
Igbo	1.15(1.13–1.18)	**0.000**	1.05(1.01–1.11)	**0.032**
Others	1.09(1.04–1.15)	**0.001**	1.01(0.98–1.04)	**0.038**
**Religion**				
Islam	Reference			
Christians	1.08(1.07–1.10)	**0.000**	0.99(0.96–1.02)	0.254
Others	1.03(0.93–1.14)	0.554	0.95(0.84–1.07)	0.201
**Marital Status**				
Never married	1.01(0.94–1.07)	0.864	+	
Living with a spouse	1.01(0.97–1.06)	0.549		
Widowed/Divorced/Separated	Reference			
**Household Wealth status**				
Low	Reference			
Middle	1.09(1.07–1.11)	**0.000**	1.02(1–00-1.04)	0.026
Richest	1.17(1.15–1.19)	**0.000**	1.03(1.01–1.06)	**0.034**
**Children ever-born**				
1 or 2 births	1.04(1.02–1.06)	**0.000**	1.01(0.98–1.04)	0.204
3 or 4 births	1.04(1.02–1.06)	**0.000**	1.01(0.98–1.03)	0.378
More than 4 births	Reference			
**Current employment status**				
Employed	1.03(1.01–1.05)	**0.001**	0.97(0.93–1.01)	0.074
**Spouse current employment status**				
Employed	1.04(1.02–1.06)	**0.000**	1.04(1.01–1.09)	**0.039**
**Place of antenatal care**				
Health facility	1.42(1.35–1.49)	**0.000**	1.37(1.29–1.44)	**0.000**
**Provider of ANC assistance**				
Skilled provider	1.19(1.16–1.22)	**0.000**	1.10(1.07–1.12)	**0.000**
**Household head sex**				
Male	1.03(1.01–1.05)	**0.016**	1.00(0.98–1.03)	0.390
**Wanted Last child**				
Then	1.03(0.98–1.07)	0.243	+	
Later	1.01(0.97–1.06)	0.576		
Never	Reference			
**Family Mobility**				
Less stable 0-4 yr	1.04(1.03–1.06)	**0.000**	1.00(0.98–1.02)	0.472
**Have health Insurance**				
Yes	1.09(1.05–1.14)	**0.000**	1.01(0.96–1.06)	0.338
**Region**				
North West	Reference			
North Central	1.07(1.04–1.1)	**0.000**	1.00(0.93–1.07)	0.462
North East	1.00(0.98–1.02)	0.933	1.01(0.94–1.08)	0.399
South East	1.16(1.13–1.19)	**0.000**	1.06(1.01–1.12)	**0.017**
South South	1.05(1.02–1.08)	**0.001**	1.00(0.92–1.08)	0.493
South West	1.12(1.1–1.15)	**0.000**	1.06(0.96–1.15)	0.123
**Random Effects**				
**Community Level**				
**Residence**				
Urban	1.09(1.07–1.11)	**0.000**	1.01(0.99–1.03)	0.154
**Community SES Disadvantage**				
Lowest	1.19(1.17–1.21)	**0.000**	1.04(1.01–1.07)	**0.023**
Middle	1.12(1.10–1.14)	**0.000**	1.01(0.98–1.05)	0.170
Highest	Reference			
**Community Unemployment rate**				
Low	1.00(0.99–1.02)	0.559	+	
**Community media barrier rate**				
Low	1.07(1.06–1.09)	**0.000**	1.01(0.99–1.03)	0.176
**Community Illiteracy rate**				
High	1.05(1.03–1.07)	**0.000**	1.03(1.01–1.04)	**0.048**
**Community Poverty rate**				
High	1.03(1.02–1.05)	**0.000**	1.01(0.99–1.04)	0.175
**State Level**				
**Rural population proportion**				
Low	1.09(1.07–1.11)	**0.000**	1.03(1.01–1.06)	**0.034**
Middle	0.91(0.97–1.01)	0.160	0.95(0.91–1.00)	0.053
High	Reference			
Model Estimates				

State level: MIRR = 1.05 (1.04–1.08), VPC (ICC) = 0.09(0.05–0.17), % Explained variation = 56.2(29.9–77.3) Community Level: MIRR = 1.02 (1.01–1.07), VPC (ICC) = 0.10(0.05–0.33), % Explained variation = 49.0(28.6–75.4)

*IRR* Incidence Rate Ratio, *MIRR* Median Incidence Rate Ratio, *CrI* Credible Interval, *VPC* Variance Partition Coefficient, *ICC* intraclass correlation, *BIC* Bayesian Information Criteria, + dropped from adjusted model

### Clustering of ANC components received

**Fig. 8:**
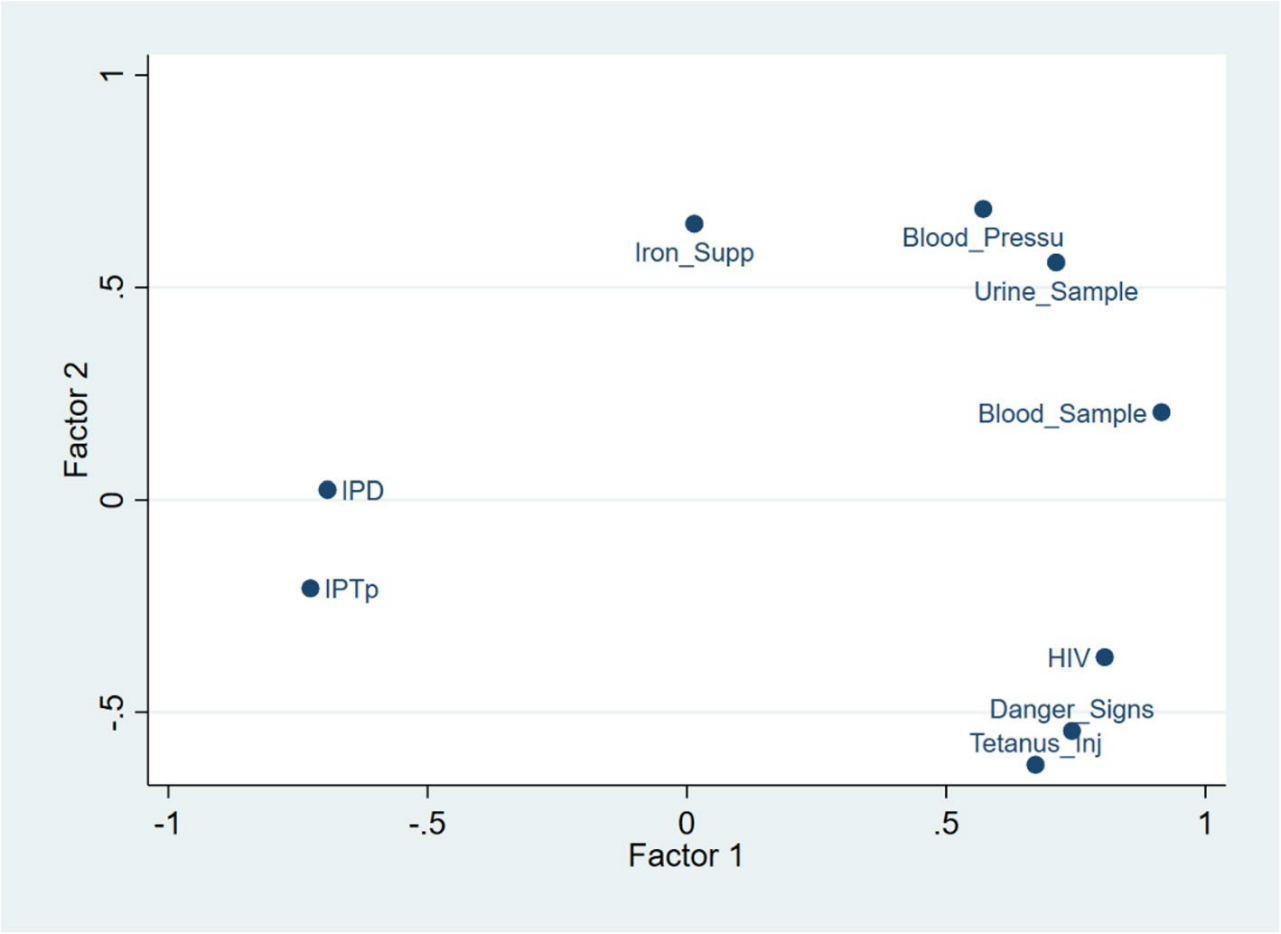
Clustering of ANC components received during the most recent pregnancy in Nigeria

Figure 8 shows the clustering of components of ANC received. It shows that pregnant women who received information on HIV, are likely to receive information on danger sign warnings and get tetanus injections while having urine tests, blood pressure and blood test formed a cluster and finally having IPD and IPTp formed another cluster. As shown, there are lower chances of having IPD and IPTp compared with the other components.

## Discussions

### Main results and comparison with existing literature

This study was designed to assess the level of ANC components offered to pregnant women in Nigeria during ANC contacts in Nigeria and the associated factors. We found that only one in every twenty pregnant women received all the 9 ANC components assessed in this study. The ANC components are having blood pressure measured, receiving a tetanus injection, having a urine test, having a blood test, being given an iron supplement, reported taking three or more doses of IPTp, IPD, and being told about danger signs and HIV/PMTCT. In comparison with the findings of Fagbamigbe et al., the proportion of women who received all ANC components offered remained low [17]. The authors assessed the quality of ANC in Nigeria using data collected in the same setting five years earlier, the authors found that only 12% of the respondents had the 7 critical components of ANC [17]. One would have expected a much higher tremendous improvement in the quality of ANC services following the rejuvenation of Nigeria's primary healthcare system in recent times through the World Bank sponsored “Nigerian State Health Investment Programme (NSHIP)” [31]. The low level is despite the well-known fact that the quality of ANC care significantly reduces mortality, morbidity, and disabilities and improves health maternal and child health outcomes [32–34]. Globally, ANC and its compliance remain the safest approach for safe motherhood [6–8].

Although at least 79% of the women received each of the 7 of the 9 components of ANC during the last pregnancy, only 24% and 22% each had IPTp and IPD respectively. Nigeria is a malaria-infested country, despite that the disease is preventable, treatable, and curable, with over 25% of the annual 25–30 million pregnant women in malaria-endemic areas in Africa [35], yet less than a quarter received the prescribed doses of IPTp. It is worrisome that only a quarter of pregnant women in Nigeria had IPTp during their last pregnancy and only 17% of women with a live birth in the 2 years preceding the survey reported having at least 3 doses of IPTp [15]. Malaria is one of the foremost public health problems in the country. Nigeria alone accounts for a quarter of the burden of malaria in Africa. This was why the Federal Ministry of Health recommended that all pregnant women should have at least three doses of IPTp in the course of each pregnancy. There is an urgent need to increase the uptake of IPTp in Nigeria. The low level of pregnant women offered IPD resonates with the little attention paid to the fight against intestinal parasites during pregnancy in Nigeria, the long clamour for its use to address this public health problem notwithstanding [36, 37]. Nigeria, just like most sub-Saharan African countries, harbours the greatest proportion of global intestinal parasitic infections largely as a result of her socioeconomic and environmental challenges [38–40].

Socio-demographic and economic characteristics pose a significant influence on the number of ANC components offered to pregnant women. In this study, similar to the findings of other studies in LMIC, various characteristics including respondents’ age, education, and economic class significantly influenced the adequacy of ANC components received [20, 41–43]. In particular, pregnant women with higher educational attainment had a higher likelihood of receiving a higher number of components of ANC relative to those with no education. Similarly, there is a higher likelihood of receiving all components among pregnant women aged 40 to 49 years relative to those aged 15 to 19 years just as those from households in the wealthiest quintile had higher chances of receiving complete ANC components. Although education mediates in the choice of place of ANC services [41, 44], and more educated women are more likely to belong to households in wealthier quintiles [29] and initiate ANC earlier [33, 45], it is not certain if different categories of women attending the same facility received different components based on their educational status. This will require further studies. Women with better-educated spouses also have significantly higher chances of receiving all the components.

Women who make decisions on their healthcare utilization alone or jointly with their spouses had significantly higher chances of getting more components than those whose spouses are the only decision-maker in women's healthcare. Our finding is in agreement with existing literature [41, 44, 46]. This ease of decision-making on women's healthcare utilization can greatly influence the timeliness of ANC service uptake, the number of ANC contacts made, and consequently, the quality of ANC services received.

Although insignificant in the multivariable model, the proportion that received all the components among the Christians nearly tripled the proportion among the Muslim women. This might be ascribed to the fact that Christians provide mission delivery and care and thereby provide the components better than others. Also, Christians may often receive double ANC service. Most times, Christian pregnant women receive ANC services from both the mission houses as well as other government facilities [47, 48]. This could have increased the quality of ANC services among Christian women than among other women. Nonetheless, the differences in the number of ANC components received across the religious groups could be largely due to the states of residence of the respondent as most Muslim women reside in the northern states while Christian women are mostly from the southern states. These differentials could be explained by the prevalent low level of education, poverty and nomadic lifestyle of the northerners. These may hinder their use of ANC and by extension, the number of components received.

Another significant variable in the receipt of a higher number of ANC components is ethnicity. A higher number of components were commoner among the Igbo women than the Hausa/Fulani women but insignificantly different when compared with the Yoruba women [33, 45]. From this study, there were wide variations in the adequacy of components received by these background characteristics. Although insignificant in the adjusted model, receipt of higher numbers of the component was higher among pregnant women who have less mobility, did not experience violence and who have health insurance. Women with health insurance have a higher likelihood of having quality ANC services. This finding points to the fact that the components are mostly offered when the ANC services are covered by insurance than when they are provided free of charge.

We found that pregnant women from the same communities have a similar likelihood of receiving similar components. Those from the least disadvantaged social-economic status communities had a preponderance of receiving a higher number of ANC components than those from the most disadvantaged communities. Pregnant women from communities with a low proportion of illiterate women also had higher chances of receiving a higher number of components just as those from states with a low rural population received higher numbers of components. The skill and distance of ANC providers to a community can greatly influence the adequacy of ANC components offered [49].

There were wide variations in the completeness of components received by background characteristics and the state of origin of the women. There is a need to overturn the inadequacies and eliminate disparities and variations in receiving all the ANC components. These variations and disparities could contribute to poor pregnancy outcomes [22]. Of significance in this study, are the type of health facility where ANC services were sourced and the skill of the ANC service provider. Pregnant women who attended institutional health care services including public and private standard hospitals and those attended to by qualified nurses and doctors had a higher level of receiving a higher number of ANC components. Similar studies have associated these factors with the quality of ANC services [22, 24, 43, 50]. This association could be ascribed to a better knowledge of the importance of the components among trained nurses, midwives, and doctors than the other unskilled providers who are usually found in non-institutional facilities [12].

We found that the number of ANC visits made during pregnancy and the timing of the first ANC visit were both significantly associated with the number of ANC components received. The earlier the initiation of ANC contacts, the higher the prevalence of having a higher number of ANC components. Fagbamigbe et al. had already suggested that early initiation of ANC contacts and having a sufficient number of contacts may increase the number of components of ANC received [2, 3]. Also, the higher the number of ANC contacts made, the higher the prevalence of having a higher number of ANC components. This is intuitive as initiating ANC contact early during the first trimester could increase the proportion of pregnant women making eight or more visits and the quality of ANC received will increase proportionately. Our finding is corroborated by findings of earlier studies that the high quality of ANC services is a direct consequence of early initiation and the sufficient number of ANC contacts [22, 24, 43, 50]. Literature is replete that ANC services significantly promote maternal and child health outcomes and by extension, reduction of maternal and child mortalities [22, 41, 51, 52].

It is striking that all the states in the Northern regions of Nigeria except Kwara and FCT had less than the national average that received all the 9 components of ANC. The average proportion who received all the 9 components in the North Central, Northeast, and North West were 5%, 3%, and 2% respectively. The prevalence in Kebbi State was 0.3% while Borno had 0.1%. Similar geographical variations in the quality of ANC have been documented [12]. States with the highest prevalence of ANC attendance had the highest prevalence of women receiving all components and vice versa. Although the prevalence is generally poor in Nigeria, the States with low ANC utilization and low prevalence of having all ANC components should take a cue from states such as Lagos, Ogun, Abia, and Anambra with better indicators. Additionally, we found clustering among having talks on HIV prevention and transmission, talk on danger signs and having tetanus injection while having urine test, blood pressure and blood test formed a cluster and finally having IPD and IPTp formed another cluster. There were distinct variabilities in the clustering of these ANC components.

### Implications for policy

There is a need to ensure that all pregnant women receive all ANC components. Stakeholders should increase supplies, train, and create awareness among ANC providers and pregnant women. States such as Sokoto should understudy what works in states such as Abia, Anambra, Enugu, and Oyo with high ANC utilization and a high level of adequate ANC components received by pregnant women. We recommend a timely initiation of ANC services alongside a sufficient number of ANC contacts. There is a need to enhance the socio-economic status of women in terms of education and ensure women have autonomous decision-making power on their health care utilization.

### Study strengths and limitations

A major limitation of this study is the use of secondary data that prevented us from a detailed assessment of health system factors. Besides, we couldn’t assess within-facility variations due to limited data. Also, our data was supplied by respondents without any way of considering facility-level data and facility-level factors occasioned by the secondary nature of the data used. The data were based mostly on respondents’ ability to recall the number of ANC contacts during pregnancy, except few cases that were found on ANC cards. A recall bias is not unlikely. Caution should be exercised when interpreting our findings. The identified factors in this study are only associated with the number of ANC components received and should not be taken for causes of the number of ANC components received as the study was only cross-sectional in design. Local Government Area (LGA) is a potential level in the multilevel analysis but couldn’t be used since LGA-level data and LGA-level characteristics are not available in the dataset.

However, our findings are generalizable as the sample was nationally representative. More so, the data source has been reported to use rigorously tested collection tools, procedures, and trained personnel on questionnaire administration. The computationally intensive methods used for data analysis guaranteed the accuracy and reliability of our estimates. This study is probably the first to explore the clustering of ANC components received by pregnant women in Nigeria.

## Conclusions

The overall prevalence of receiving all the 9 components of ANC received during ANC contacts in Nigeria is poor. There is a need to ensure that all pregnant women receive an adequate and optimal number of ANC components. Different factors influenced the number of ANC components received. Notable among them are the institution and skill of the ANC provider, education, and social-economic status of the women. The study shows a strong relationship between the timeliness of ANC initiation and having a minimum of 8 ANC contacts as they both increased the adequacy of ANC components received during the contacts. Although there are some states with high ANC utilization but inadequate ANC components. Our study suggested that early initiation of ANC during the first trimester and having the recommended number of ANC contacts are critical to having all ANC components, which in turn ensures that a pregnant woman and her unborn child take optimal advantage of ANC services.

## Supplementary Information

**Additional file 1: Supplementary Table.**A. Distribution of having all ANC component received during the most recent pregnancy by States and regions in Nigeria. B. The BIC and ICCs of the levels of the different models considered. 

## Data Availability

The data supporting this article is available on request at www.dhsprogram.com and contact Bridgette Wellington, the Data Archivist.

## Abbreviations

aIRR: Adjusted Incidence Risk Ratio Ratio
ANC: Antenatal care
BIC: Bayesian Information Criteria
BP: Blood Pressure
BS: Blood Test
CI: Confidence Interval
EAs: Enumeration areas
FCT: Federal Capital Territory
IRB: Institutional Review Board
IPD: Intestinal parasite drugs
IPTp: Intermittent preventive treatment in pregnancy
ICC: Intraclass Correlation Coefficient
IRS: Iron Supplement
LGAs: Local government areas
LMIC: Low and Middle-Income Countries
NDHS: Nigeria Demographic Health Survey
PRMM: Pregnancy-related maternal mortality
PSU: Primary sampling unit
SES: Socioeconomic and
SSA: Sub-Sahara Africa
SDG: Sustainable development goals
TET: Tetanus Injection
UNICEF: United Nations Children’s Fund
UNFPA: United Nations Population Fund
US: Urine Test
VPC: Variance Partition Coefficient
WHO: World Health Organization
