# Supplementary material for: A multilevel Bayesian Markov Chain Monte Carlo Poisson modelling of factors associated with components of antenatal care offered to pregnant women in Nigeria
Source: BMC Health Serv Res. 2023 Jul 5;23:728. doi: 10.1186/s12913-023-09710-2 (PMC10320930; doi:10.1186/s12913-023-09710-2)
Supplement: Supplementary file 1 — Additional file 1: Supplementary Table.A. Distribution of having all ANC component received during the most recent pregnancy by States and regions in Nigeria. B. The BIC and ICCs of the levels of the different models considered. [file 12913_2023_9710_MOESM1_ESM.docx]

**A multilevel Bayesian Markov Chain** **Monte Carlo Poisson modelling of factors associated with components of antenatal care offered to pregnant women in Nigeria**

Omon Stellamaris Fagbamigbe^a^, Olugbenga Sunday Olaseinde^b^, Oluwasomidoyin O. Bello^c^, Vincent Setlhare^d^, Jackline Mosinya Nyaberi^e^, Anthony Ike Wegbom^f^, Ayo Stephen Adebowale^g,h^, *Adeniyi Francis Fagbamigbe^g,i,j^

^a^Portsmouth Business School, Faculty of Business and Law, University of Portsmouth, United Kingdom

^b^Department of Sociology, Adekunle Ajasin University, Akungba Akoko, Nigeria

^c^Department of Obstetrics and Gynaecology, College of Medicine, University of Ibadan, Ibadan, Nigeria ^f^ ^d^Department of Family Medicine and Public Health, Faculty of Medicine, University of Botswana, Gaborone, Botswana.

^e^Department of Environmental Health and Disease Control, Jomo Kenyatta University of Agriculture and Technology, Nairobi, Kenya.

^f^Department of Public Health Sciences, College of Medical Sciences, Rivers State University, Port Harcourt, Nigeria.

^g^Department of Epidemiology and Medical Statistics, College of Medicine, University of Ibadan, Ibadan, Nigeria

^h^Population Health and Research Entity, North-West University, Mafikeng, South Africa

^i^Health Data Science Unit, Division of Population and Behavioural Science, School of Medicine, University of St Andrews, St Andrews, United Kingdom

^j^Institute of Applied Health Sciences, School of Medicine, Medical Sciences & Nutrition, University of Aberdeen, Aberdeen, United Kingdom

*Correspondence: [franstel74@yahoo.com; fadeniyi@cartafrica.org](mailto:franstel74@yahoo.com;%20fadeniyi@cartafrica.org); +2348061348165; ORCID: 0000-0001-9184-8258

Omon Stellamaris Fagbamigbe: omonstellamaris@yahoo.com

Olugbenga Olaseinde: [gbengaseinde@gmail.com](mailto:gbengaseinde@gmail.com)

Oluwasomidoyin O. Bello: bellodoyin@yahoo.com

Vincent Setlhare : [setlharev@ub.ac.bw](mailto:setlharev@ub.ac.bw)

Jackline Mosinya Nyaberi: [jnyaberij@gmail.com](mailto:jnyaberij@gmail.com)

Ayo S. Adebowale: adehamilt2008@yahoo.com

Adeniyi Francis Fagbamigbe: [franstel74@yahoo.com; fadeniyi@cartafrica.org](mailto:franstel74@yahoo.com;%20fadeniyi@cartafrica.org)

Supplementary Table A: Distribution of having all ANC component received during the most recent pregnancy by States and regions in Nigeria

| **Region** | **State** | **Had ≥1 ANC Contacts (%)** | **n** | **All 9** |
| --- | --- | --- | --- | --- |
| North Central |  | **72.2** | **1,420** | **4.5** |
|  | Plateau | 75.9 | 203 | 2.6 |
|  | Kogi | 82.2 | 147 | 0.4 |
|  | Niger | 59.2 | 271 | 0.8 |
|  | Nasarawa | 77.6 | 185 | 4.8 |
|  | Benue | 74.6 | 356 | 8.3 |
|  | FCT, Abuja | 87.7 | 91 | 3.4 |
|  | Kwara | 74.6 | 167 | 8.6 |
| North East |  | **71.5** | **2,142** | **3.1** |
|  | Borno | 62.4 | 350 | 0.1 |
|  | Taraba | 79.6 | 280 | 1.3 |
|  | Yobe | 69.8 | 417 | 2.1 |
|  | Bauchi | 66.9 | 493 | 2.4 |
|  | Gombe | 74.5 | 251 | 3.5 |
|  | Adamawa | 84.5 | 351 | 9.3 |
| North West |  | **63.7** | **3,772** | **1.5** |
|  | Kebbi | 52.1 | 281 | 0.3 |
|  | Kano | 83.6 | 1,106 | 0.6 |
|  | Katsina | 53.0 | 635 | 3.2 |
|  | Kaduna | 70.0 | 787 | 1.4 |
|  | Zamfara | 36.3 | 187 | 0.0 |
|  | Sokoto | 46.9 | 176 | 2.7 |
|  | Jigawa | 79.5 | 600 | 2.1 |
| South East |  | **96.2** | **1,479** | **8.3** |
|  | Ebonyi | 94.4 | 349 | 6.4 |
|  | Enugu | 96.2 | 222 | 2.4 |
|  | Imo | 97.5 | 277 | 6.7 |
|  | Anambra | 96.6 | 456 | 4.5 |
|  | Abia | 96.2 | 175 | 31.7 |
| South South |  | **81.1** | **1,111** | **5.8** |
|  | Cross River | 84.0 | 132 | 6.5 |
|  | Edo | 90.0 | 165 | 0.7 |
|  | Bayelsa | 42.3 | 47 | 6.1 |
|  | Rivers | 87.6 | 360 | 4.6 |
|  | Akwa Ibom | 80.7 | 206 | 11.4 |
|  | Delta | 76.7 | 201 | 5.7 |
| South West |  | **94.1** | **1,945** | **12.2** |
|  | Osun | 98.8 | 236 | 3.6 |
|  | Lagos | 95.4 | 691 | 10.4 |
|  | Ekiti | 93.1 | 145 | 5.4 |
|  | Oyo | 87.9 | 402 | 19.6 |
|  | Ondo | 95.4 | 186 | 1.5 |
|  | Ogun | 96.2 | 285 | 23.5 |
| **Total** |  | **75.2** | **11,867** | **5.1** |

Supplementary Table B: The BIC and ICCs of the levels of the different models considered.

|  | Null model | Individual-level alone | Community-level alone | State-level alone | All levels included |
| --- | --- | --- | --- | --- | --- |
| ICC* |  |  |  |  |  |
| Individual | 1 | 1 | 1 | 1 | 1 |
| Community | 0.34(0.29-0.44) | 0.34(0.30-0.45) | 0.19(0.13-0.26) | 0.29(0.27-0.36) | 0.10(0.05-0.33) |
| State | 0.14(0.12-0.17) | 0.19(0.12-0.26) | 0.21(0.13-0.28) | 0.06(0.01-0.01) | 0.09(0.05-0.17) |
| BIC | 45898.6 | 43215.9 | 44158.1 | 44211.7 | 41927.3 |
| ICC Intraclass Correlation Coefficient BIC Bayesian Information Criteria *computed as ratio of the variability in the community and state levels compared with the individual levels | | | | | |
